# Ingestion of oxygenated water enhances lactate clearance kinetics in trained runners

**DOI:** 10.1186/s12970-017-0166-y

**Published:** 2017-03-29

**Authors:** Neil Fleming, Jeremiah Vaughan, Matthew Feeback

**Affiliations:** 1 Human Performance Laboratory, Department of Anatomy, Trinity College Dublin, Dublin, Ireland; 2 Department of Exercise Physiology, Kent State University, Kent, Ohio USA; 3 Movement Science Department, Grand Valley State University, Allendale, Michigan USA

**Keywords:** Hydration, Running, Recovery

## Abstract

**Background:**

Drinks with higher dissolved oxygen concentrations have in recent times gained popularity as a potential ergogenic aid, despite a lack of evidence regarding their efficacy. The aim of this study was to assess effects of ingestion of an oxygen supplement (OS) on exercise performance and post-exercise recovery in a group of trained runners.

**Methods:**

Trained male runners (*n =* 25, mean ± SD; age 23 ± 6 years, mass 70 ± 9 kg, BMI 21.9 ± 2.7 kg.m^−2^ VO_2_max 64 ± 6mL.kg^−1^.min^−1^), completed a randomised double blinded, crossover study to assess the effect of ingestion of OS solution on exercise performance and recovery. Trials consisted of a 30min rest period, 5min warm-up, a 5000m treadmill time-trial, and a 30min passive recovery. Participants ingested 6x15mL of either OS or a taste matched placebo during the trials (3 during the rest phase, 1 during exercise and 2 during the recovery). Muscle tissue O_2_ saturation was measured via near infrared spectroscopy. Blood lactate concentrations were measured prior to, mid-way and directly after the finish of the 5000m time trials and every 3-min during the post-exercise recovery.

**Results:**

Ingestion of OS did not improve exercise performance. No significant differences were observed for muscle tissue O_2_ saturation at any time-points. However, lactate clearance was significantly improved during recovery in the OS trials. Both AUC (109 ± 32 vs. 123 ± 38 mmol.min, *P <* 0.05, d = 0.40) and lactate half-life (λ) (1127 ± 272 vs. 1223 ± 334 s, *P <* 0.05, d = 0.32) were significantly reduced.

**Conclusions:**

Despite no evidence of improved exercise performance, ingestion of OS did enhance post-exercise recovery via increased lactate clearance.

## Background

The study of oxygen supplementation dates back to the 1940’s, when high altitude climbers fought - and in many cases died—to plant their nation’s flag on the world’s highest peaks. Climbers used bottled oxygen to supplement their breathing in order stay alive in the extreme environment above 8000m, known as “the death zone”. Many studies from this period documented the physiological effects of inspiring higher concentrations of oxygen during exercise. Such effects include increased arterial oxygen saturation [1], decreased pulmonary ventilation [2], lower submaximal heart rate and blood lactate concentration [3], and increased VO_2_max [4].

More recently, commercially available drinks advertising high concentrations of dissolved O_2_ have become popular. Despite anecdotal reports from athletes, few controlled studies have been conducted and therefore the ergogenic effects of these drinks remains questionable [5]. Of the limited studies which have examined the effects of oxygenated water, most have reported no effect on aerobic performance [6–10]. The majority of these studies have used VO_2_ either at sub-maximal or maximal intensity as a measure of aerobic performance [6–9]. However, it is not immediately clear why these researchers would expect to see any change in VO_2_ following ingestion of supplemental oxygen. VO_2_ is calculated using the Haldane Transformation which assumes that O_2_ consumption is equal to the difference between inspired O_2_ and expired O_2_. Since ingested O_2_ is not accounted for in this equation, any additional O_2_ which is theoretically utilized by the working muscles would not be detected using pulmonary gas measurement.

The primary criticism put forward by skeptics of oxygenated drinks is that ingested O_2_ is not readily diffused across the gastro-intestinal tract [6, 9, 11]. However, two studies have demonstrated that high-concentration oxygen solutions are capable of diffusing O_2_ into the bloodstream, albeit into the hepatic portal vein in rabbits [12] and kittens [13]. Neither study assessed whether this gas diffusion altered the systemic or peripheral arterial saturation, and so extrapolating a potential ergogenic effect at a muscular level is pre-mature. To date, no study has verified if the human gastro-intestinal tract has the potential to absorb O_2_ into the hepatic portal vein. Previous human studies have used pulse oximetry [5, 10, 14] or blood gas analysis [6, 8] to quantify systemic O_2_ concentrations with most reporting no change in oxygen saturation. However, muscle tissue O_2_ saturation has yet to be determined.

The majority of studies examining the ergogenic effects of oxygenated water report that exercise performance is not improved [6–10]. Only one study, which recruited higher trained athletes, reported improvements in performance and increased O_2_ saturation ([14] abstract only). However, an interesting effect of oxygenated water has been observed in two separate studies [7, 8]. Despite both studies reporting no improvement in performance, the authors did observe lower maximal lactate concentration and enhanced lactate clearance post-exercise. In both studies, this finding was statistically significant and it is curious that neither group discussed the potential implications of such a finding on recovery. In addition, neither study tracked lactate clearance kinetics for longer than 6 min, so the full effect has not been established.

Based on the limited published literature examining ingestion of oxygen supplements (OS), it appears that a more comprehensive evaluation is warranted. In addition, the effects on lactate clearance kinetics require further examination, since the two studies which previously measured this variable showed a significant effect [7, 8]. The primary aim of this study was to investigate if ingesting OS had an ergogenic effect on exercise performance. A secondary aim was to assess its effect on muscle O_2_ saturation and lactate concentration before, during and after exercise.

## Methods

### Study design

A cohort of 25 male collegiate level distance runners (see Table 1) performed double-blinded, placebo controlled trials in a counterbalanced cross-over design in order to assess the effect of OS ingestion on performance, muscle O_2_ saturation and blood lactate kinetics. Subjects reported a minimum of 200 min.week^−1^ of running and were excluded if they had any medical condition that prevented them training for more than 7 consecutive days in the previous 6 months. All subjects attended the laboratory on three occasions. During the first visit, they were familiarized with all procedures and equipment. If satisfied, informed consent was obtained and pre-trial physiological data collected. The second and third visits comprised of ingestion of either OS or placebo, followed by a 5000m self-paced treadmill time-trial. Ethical approval for this study was granted by Indiana State University’s Institutional Review Board and informed consent was obtained from all subjects prior to data collection.

**Table 1:** Anthropometric and physiological characteristics of the group

Variable	Group mean ± SD
Age (yr)	23 ± 6
VO_2_max (mL.kg^−1^.min^−1^)	63.8 ± 5.7
Height (m)	1.78 ± 0.07
Mass (kg)	69.9 ± 8.8
Percentage Body Fat (%)	10.4 ± 3.5

### Exercise protocol

During the initial visit, subjects performed a maximal incremental test, in order to quantify VO_2_max. Starting velocity for the test was 10km.h^−1^ with an increase of 1km.h^−1^ every 3-min until volitional failure was reached. Pulmonary gas exchange data was collected throughout the test using a Parvomedics TrueOne 2600 analyser. VO_2_max was defined as the maximal VO_2_ recorded during any 15-s interval.

**Fig. 1:**
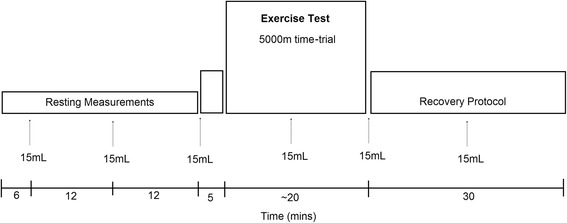
Diagram of the protocol timeline. 5000m time-trials were performed in a randomized order (placebo vs. OS)

During visits 2 and 3, subjects performed a self-paced 5000m time-trial on a treadmill (Woodway Forefront) following ingestion of OS or a taste-matched placebo. All subjects were instructed not to eat or consume caffeine in the 120-min prior to testing, in order to ensure appropriate gastric emptying prior to ingestion of solutions and to reduce possible confounding effects that caffeine might have on exercise performance. Repeat tests were performed at the same time of the day in order to minimize the effect of circadian variability. Subjects ingested a series of 6 × 15mL volumes of either OS or taste matched placebo, at fixed time-points before, during and after the 5000m time trial (see Fig. 1).

The composition of 15mL of OS was ASO® solution (Activate Stabilized Oxygen), a registered dietary oxygen supplement for human consumption. The ingredients to ASO® are the following:

- Distilled water: (62.04%)
- Dissolved O_2_ (in molecular O_4_ form): (35.00%)
- Salt & trace elements: (2.96%)

The taste-matched placebo comprised of 0.6mg of NaCl added to 15mL of distilled water. Taste testing was carried out prior to the initiation of the study. 50 students and faculty were asked to taste both solutions and decide which one they thought was the OS solution, with correct responses no better than chance. Following completion of both exercise trials, subjects were asked if they could differentiate between drinks based on taste. 11 of the subjects stated they could taste a difference between drinks but were unable to determine which drink contained OS. The remaining 14 subjects could not taste any difference between drinks.

Upon completion of the 30-min seated rest period, subjects performed a 5min warm-up at a self-selected pace. They then performed a self-paced 5000m time trial. Information on elapsed time and distance covered were provided continuously throughout the test via the treadmill monitor. Subjects were instructed to run the 5000m distance as fast as they possibly could. They were free to increase and decrease the treadmill velocity as necessary during the time-trial, but all subjects were encouraged to complete the distance as fast as possible. The 5000m distance was selected because the duration and intensity are such that the aerobic system is near maximally stressed. It has previously been reported that well-trained runners utilise an average of 94% of their VO_2_max when running a simulated 5000m race on a treadmill [15]. In addition, previous studies have demonstrated that test-restest reliability for 5000m time-trials is higher than submaximal endurance trials of a similar intensity [16]. Upon completion of the test, participants began a 30min passive recovery in a seated position (see Fig. 1).

### Blood lactate data

Capillary blood samples were collected from the middle finger of the left hand using aseptic techniques, to measure blood lactate concentrations before, during and after exercise. Samples were collected immediately before, at the mid-point and immediately upon completion of the time-trial. Additional samples were collected at 3min intervals during the recovery. A 7μL sample was injected into an Analox GM7 metabolic analyzer. A new Teflon membrane was installed in the analyzer prior to the initiation of data collection. Test-retest analysis of the membrane was carried out via 10 repeat measures of 8mMol.L^−1^ lactate standard. The CV for this protocol was 0.6%, which falls within the acceptable range of 0–1%, set by the manufacturer. The lactate analyser was calibrated prior to each exercise test (to a resolution of ±0.1mmol.L^−1^), using 8mmol.L^−1^ lactate standard.

### Oxygen saturation

Muscle tissue oxygen saturation was measured at 10Hz continuously from the right *Rectus Femoris* using a portable NIRS monitor (Portamon MkII, Artinis). The right *Rectus Femoris* was chosen over the *Vastii* muscles due to the observation of more stable data with less motion artifact during pilot testing in running. Since the midpoint of the *Rectus Femoris* is located more proximal to the hip joint than either of the *Vastii* muscles, the sensor movement was lower with this muscle. The recording site was shaved and cleaned with isopropyl alcohol prior to application of the NIRS sensor. In addition, the recording site was marked with permanent marker in order to ensure correct sensor placement on repeat visits. Data were recorded continuously throughout the rest, exercise and recovery phases and reported as a combined tissue saturation index (%TSI). A 6-min control period prior to fluid ingestion allowed for the measurement of baseline %TSI. Changes in %TSI were then averaged for each 3-min interval and normalized to baseline for each trial. This method of estimating tissue oxygen saturation has been validated and used for the last 10 years as a measure of muscle oxygenation [17].

### Statistical analysis

All statistical tests were performed using Graphpad Prism Version 6.0 (GraphPad Software, CA, USA). Performance was quantified as time to completion (TTC) of the 5000m time-trial. Lactate clearance kinetics were quantified during recovery by measuring the area under the curve (AUC) and the time taken to reduce the peak lactate concentration by 50% (λ), also known as lactate half-life. A 4th order polynomial was fitted over the 10 blood lactate concentrations measured following completion of the time-trial. AUC and λ were subsequently computed using Matlab (V7.14 R2012a, Mathworks, MA, USA). All data sets were initially tested for normality via Kolmogorov-Smirnov tests. For comparison of data across time between the two drink trials, a 2-factor ANOVA (drink x time) with 1 repeated measure (drink) was used. Bonferoni *post-hoc* tests quantified significance where identified. For variables which were independent of time (TTC, final lactate, λ, AUC), paired Student’s T-tests were performed, with statistical significance inferred at *P <* 0.05. Where statistically significant differences between drinks was observed, effect sizes (Cohen’s D) were computed, with >0.2 indicating a small effect, >0.5 a moderate effect and >0.8 a large effect.

## Results

### Performance data

Group anthropometric and physiological data are presented in Table 1. The group mean (± SD) TTC data were 1096 ± 80 and 1102 ± 93 s, for OS and placebo trials, respectively. The group performed an average of 6 s faster during the OS trials; however, this difference did not attain statistical significance.

### Blood lactate data

**Fig. 2:**
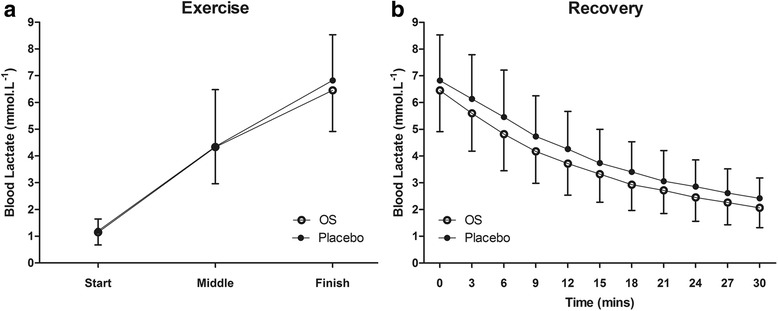
Group mean (SD) lactate concentration during exercise (**a**) and recovery (**b**). Data were collected immediately prior to the start, at the mid-point (2500m) and immediately following the finish, and every 3-min during the 30min recovery

**Fig. 3:**
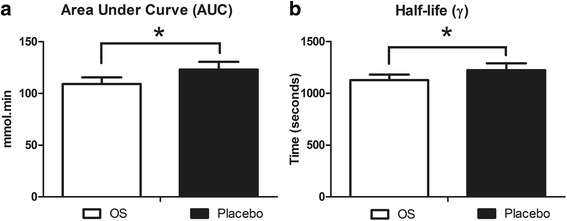
Group mean (SD) lactate clearance kinetics during recovery, measured as area under the cure (**a**) and half-life (**b**). Asterisk infer significant difference between OS and Placebo trials (*, *P <* 0.05)

A significant time effect was observed for lactate concentrations both during exercise and recovery (*P <* 0.001). This time effect was statistically significant in both OS and placebo trials. Group mean lactate concentrations were lower during the OS trials at the mid-point and finish of time-trials, and at every time-point during recovery (see Fig. 2), however this effect failed to attain statistical significance. Final lactate concentrations at the end of the 5000m time-trial were 6.5 ± 1.5 vs. 6.8 ± 1.7 mmol.L^−1^ for OS and placebo trials respectively. Lactate clearance kinetics were significantly different comparing OS and placebo trials. Both AUC (109 ± 32 vs. 123 ± 38 mmol.min, *P <* 0.05, d = 0.40; see Fig. 3a) and λ (1127 ± 272 vs. 1223 ± 334, *P <* 0.05, d = 0.32; see Fig. 3b) were significantly reduced during the OS trials, indicating enhanced lactate clearance.

### Oxygen saturation

**Fig. 4:**
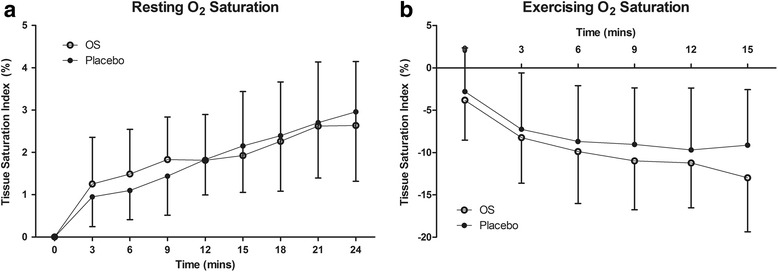
Group mean ± SD muscle tissue saturation data during the rest (**a**) and exercise (**b**) phases. All data were normalized to 6-min baseline (prior to ingestion of any fluid). The horizontal axis indicates the time after baseline in the rest phase and the time-trial in the exercise phase

During the rest period, tissue O_2_ saturation significantly increased (*P <* 0.001, see Fig. 4a) across time. This time effect was statistically significant in both OS and placebo trials and is most likely due to reduced cardiovascular stress and venous pooling associated with sitting in a chair for 30min. During exercise, there was a significant reduction in tissue O_2_ saturation across time (*P <* 0.001, see Fig. 4b), however no differences between drink trials were observed. Tissue O_2_ saturation did appear lower during OS trials however the differences to placebo were not significant (see Fig. 4b).

## Discussion

The main finding from the current study is that ingestion of OS resulted in a significant improvement in post-exercise lactate clearance. However, this apparent enhancement in lactate metabolism was not detectable during exercise and did not yield an improvement in overall performance. Additionally, there was no evidence of any increase in tissue or systemic oxygen saturation measured via pulse-oximetry and NIRS. It is possible that increased oxygen saturation at a hepatic level resulted in greater lactate metabolism by the liver, however further research is required in order to test this hypothesis.

### Lactate clearance

It is generally accepted that high intensity exercise results in significant production of lactate within the muscle [18] and this accumulation of lactate—or the associated muscle acidosis—is a major determinant of fatigue [19, 20]. Several studies have demonstrated that enhanced blood lactate clearance via active recovery, improves subsequent exercise performance [21, 22]. The current results suggest that ingestion of OS facilitates more rapid clearance of lactate and could therefore enhance recovery from exercise. Both the half-life and AUC data were significantly lower during recovery in the OS compared to placebo trials (see Fig. 3). To the best of our knowledge, this study is the first to evaluate the effects of OS on post-exercise lactate kinetics over a 30 min time course. Several other studies have also reported similar effects of OS ingestion on lactate concentrations—albeit over a shorter time course. Leibetseder, Strauss-Blasche [7] reported significantly lower lactate concentrations at the end of maximal incremental exercise. McNaughton, Kenney [8] also reported significant reductions in lactate concentrations. In this case, ingestion of OS resulted in significantly lower lactate concentrations at 0 and 3min after completion of a 15min cycling time-trial. The current findings represent further evidence of the effects of OS ingestion on blood lactate kinetics.

### Physiological mechanisms

It is tempting to suggest that enhanced delivery of O_2_ to the working muscles may explain the improvements in lactate kinetics. However, no differences in tissue or peripheral O_2_ saturation were observed before, during or after exercise. This finding is in agreement with the majority of studies examining O_2_ saturation either via pulse-oximetry [10] or blood gas analysis [6, 8, 9]. The most likely explanation for these results is that an increase in hepatic metabolism via the Cori cycle resulted in greater lactate clearance. Next to the kidneys, the liver is the most important human tissue for net lactate clearance at rest [23]. This is evident in the use of lactate clearance kinetics as a reliable clinical indicator of liver transplant success [24]. The metabolic rate of liver cells is directly influenced by surrounding oxygen concentration [25, 26], with increased metabolism of lactate occurring at higher O_2_ concentrations. Previous studies of O_2_ diffusion across the gastro-intestinal tract have reported significant increases in O_2_ concentration within the hepatic portal vein [12, 13]. While these animal studies are not directly comparable with human physiology, they nonetheless demonstrate that enhanced O_2_ delivery to the liver is at least theoretically possible following OS ingestion in humans. Increased oxygen delivery to the liver via the hepatic portal vein would therefore enhance blood lactate clearance. A similar physiological effect has also been observed in liver’s metabolism of alcohol in humans. Several studies have recently reported that oxygenated alcohol beverages resulted in increased rates of blood alcohol clearance [27, 28]. Based on the combined evidence from animal, hepatocyte and human alcohol studies, we hypothesize that enhanced lactate clearance following OS ingestion is potentially mediated by increased hepatic metabolism; however this hypothesis can only be tested via *in-vivo* measurement of liver O_2_ concentrations and/or liver enzyme activity.

### Applications to performance

Many sports such as track sprinting, cycling, swimming, and rowing require the athlete to perform on more than one occasion during a single day. The ability to clear lactate more efficiently and hence recover faster in early rounds of competition is of benefit to such athletes [22]. Lactate clearance is also of importance in several team based sports which involve intermittent exercise and recovery. For example, the sport of basketball involves continuous flow of play with players performing high intensity movements on average every 21 s [29]. However, basketball players receive regular recovery during the game via substitution, time-outs and breaks at each quarter. Despite these periods of recovery, it has been shown that players compete with average circulating blood lactate concentrations of 6.8 mmol.L^−1^ throughout the game [29]. Any intervention which enhances the clearance of lactate during a player’s recovery would therefore likely improve overall performance in such sports.

### Study limitations

The main limitation of the current study is that the protocol was not designed to elicit maximal lactate concentrations. A 5000m time-trial was used, in order to assess if OS improved performance in an activity that places high demands on the oxidative energy system. While this near-maximal aerobic exercise did result in moderate accumulation of blood lactate, high-intensity repeated intermittent sprint exercise would place greater demands on the anaerobic energy systems and thus result in substantially higher lactate concentrations [30]. Based on the current results, OS ingestion may be of greater benefit during and after anaerobic exercise, especially activities which result in large accumulation of blood lactate. Secondly, the assessment of subsequent exercise performance following a period of recovery was not assessed. Therefore our statement that enhanced lactate clearance can improve subsequent performance is based on previous work [21, 22] and not the current results. Future studies should focus on the possible ergogenic effects of OS ingestion during high-intensity anaerobic exercise and/or post-recovery performance. Finally, no direct measurement of tissue specific O_2_ concentration or liver enzymatic activity was made. Our hypothesis that OS ingestion may enhance hepatic metabolism of lactate would require direct measurement of hepatic O_2_ consumption or enzymatic activity. Further research is necessary in order to elucidate the physiological mechanisms underlying the enhanced lactate clearance observed in this study.

## Conclusions

Ingestion of OS resulted in enhanced post-exercise lactate clearance following a 5000m time-trial in trained distance runners. However, no improvements in performance or lactate kinetics during exercise were observed. The current findings may have important implications for optimizing post-exercise recovery strategies for athletes. Further research is warranted, in order to elucidate the physiological mechanisms underlying the apparent enhancement in lactate clearance kinetics.
